# Co-delivery of carboplatin and paclitaxel *via* cross-linked multilamellar liposomes for ovarian cancer treatment†
†Electronic supplementary information (ESI) available. See DOI: 10.1039/c7ra01100h



**DOI:** 10.1039/c7ra01100h

**Published:** 2017-04-03

**Authors:** Xiaoyang Zhang, Yarong Liu, Yu Jeong Kim, John Mac, Rachel Zhuang, Pin Wang

**Affiliations:** a Mork Family Department of Chemical Engineering and Materials Science , University of Southern California , 3710 McClintock Ave. , RTH509 , Los Angeles , CA 90089 , USA . Email: pinwang@usc.edu ; Fax: +1-213-740-8053 ; Tel: +1-213-740-0780; b Department of Pharmacology and Pharmaceutical Sciences , University of Southern California , Los Angeles , CA 90089 , USA; c Department of Biomedical Engineering , University of Southern California , Los Angeles , CA 90089 , USA

## Abstract

Carboplatin (CPT) and paclitaxel (PTX) used in combination is one of the most effective treatments for ovarian cancer. However, the traditional combination methods used to co-administrate CPT and PTX showed limited clinical efficacy due to their distinct pharmacokinetics. Although much effort has been devoted to developing nanoparticles capable of encapsulating drugs with different lipophilicites, co-delivery of carboplatin with paclitaxel by a single nanoparticle has rarely been reported. Here, we encapsulated and delivered this drug combination to ovarian cancer cells at a controlled ratio by a previously reported crosslinked multilamellar liposome vesicle (cMLV). A 1 : 1 CPT/PTX molar ratio for cMLVs (CPT/PTX) combination treatment was found to induce the strongest anti-tumor synergism and to target ALDH+ cancer stem cells (CSC) *in vitro*. Moreover, we demonstrated that this co-encapsulation strategy reduced systemic cytotoxicity and resulted in a stronger anti-tumor effect when compared to free drug combinations and individual drug-loaded cMLVs in an OVCAR8 ovarian cancer xenograft mouse model. Thus, this study suggests a potentially promising combination therapy for ovarian cancer in clinical practice.

## Abstract 2

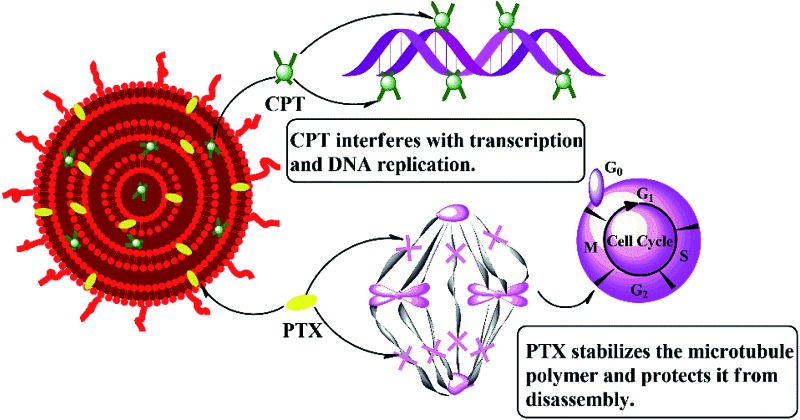
Cross-linked multilamellar liposomes offer an approach to achieve combinatorial delivery of hydrophobic paclitaxel and hydrophilic metallic carboplatin at a synergistic ratio to treat ovarian cancer.

## Introduction

Ovarian cancer is the sixth most common cancer and the seventh most common cause of cancer deaths in women.^1^ Currently, chemotherapy based on platinum drugs (carboplatin or cisplatin) in combination with paclitaxel is a standard treatment for patients with ovarian cancer,^2^ because platinum drugs and paclitaxel trigger different apoptosis signaling pathways in ovarian cancer cells.^3–5^ Moreover, platinum drugs trigger cell apoptosis by crosslinking DNA,^6^ whereas paclitaxel does so by inducing cell cycle arrest at the G2/M phase *via* binding to the beta-tubulin subunit of microtubules. Recent work has proven that the co-administration of platinum drugs with paclitaxel results in a synergistic effect in cancer treatment due to their distinct mechanisms of action.^7^ In this study, carboplatin was co-administered with paclitaxel because of its lack of renal toxicity, unlike the widely used cisplatin.^8^ Furthermore, for a comparable level of treatment effectiveness, it has been determined that the combination of carboplatin and paclitaxel yields a more tolerable quality of life for patients than the combination of cisplatin and paclitaxel. Therefore, the combination of carboplatin and paclitaxel should be regarded as a very important regimen for treating patients with ovarian cancer^9^ and even platinum-sensitive, recurrent ovarian cancer.^1^

However, the clinical results are hindered by the distinct pharmacokinetics and biodistributions of combined carboplatin and paclitaxel due to their different lipophilicities. Paclitaxel is highly hydrophobic and must be administered with a combination of dehydrated alcohol and Cremophor EL (polyoxyethylated castor oil) as an adjuvant, which can lead to serious side effects including neurotoxicity, nephrotoxicity and hypersensitivity reactions.^10^ Moreover, this administration method may hinder the accumulation of paclitaxel within tumors and compromise its *in vivo* efficacy due to its poor pharmacodynamic (cytochrome P450 metabolism) and pharmacokinetic profiles (*t*_1/2_ in human: 2.09 h).^11^ In contrast, carboplatin is eliminated and metabolized much slower *in vivo* because of its heavy metal backbone,^12^ which could lead to long-term. However, the biotransformations of carboplatin, which form in the bloodstream after hydrolysis and binding to plasma proteins in the plasm, are directly associated with its acute and chronic hematopoietic toxicity, hepatotoxicity, and neurotoxicity.^13^ Thus, a more effective paclitaxel/carboplatin combination therapy is needed to administer paclitaxel without any harmful organic solvents, improve its bioavailability, and increase its exposure to tumor cells, while simultaneously minimizing the acute and cumulative long-term chronic toxic side effects of carboplatin. This can be achieved by encapsulating both drugs in a nanoparticle drug delivery system that allows for the pharmacokinetic and pharmacodynamics behaviors of both drugs to be determined by the pharmacokinetics and pharmacodynamics of the nanoparticles.

Over the past few decades, significant effort has been devoted to developing nanotechnology for drug delivery since it allows for targeted drug delivery to tissues of interest and therefore, a significant decrease in toxic, off-target side effects.^14–17^ A variety of nanocarriers have been developed for the delivery of either paclitaxel or carboplatin.^18–20^ However, encapsulating these two drugs, which have distinct physiochemical properties, into a single nanocarrier remains challenging. Even though a few studies of the co-delivery of cisplatin with paclitaxel have been published recently,^21–23^ research on the co-delivery of carboplatin with paclitaxel has rarely been reported.

In this study, we investigated whether the cMLVs reported in our previous studies^24,25^ could achieve the synergistic combinatorial delivery of hydrophobic paclitaxel and hydrophilic carboplatin. Both paclitaxel and carboplatin achieved a high level of encapsulation efficiency and a controlled release profile in cMLVs. The flow cytometry and cell cytotoxicity results determined a synergistic drug ratio, which was further used in ovarian cancer mouse models. Furthermore, *in vivo* studies revealed that the co-delivery of paclitaxel and carboplatin *via* cMLVs could induce a potent antitumor effect while decreasing the systemic toxicity and out-performing free drug combination and single drug-loaded cMLVs. These results further demonstrate the potential of cMLVs as a novel drug delivery system for the co-localized delivery of drug combinations in cancer therapy.

## Experimental

### Cell lines, reagents and mice

OVCAR8 and NCI/ADR-RES cell lines were kindly provided as gifts by Dr Nouri Neamati (University of Southern California, School of Pharmacy, and Los Angeles, CA) and maintained in RPMI-1640 supplemented with 10% fetal bovine serum (FBS) and 2 mM of l-glutamine in a 5% CO_2_ environment.

All lipids were obtained from NOF Corporation (Japan): 1,2-dioleoyl-*sn*-glycero-3-phosphocholine (DOPC), 1,2-dioleoyl-*sn*-glycero-3-phospho-(10-rac-glycerol) (DOPG), and 1,2-dioleoyl-*sn*-glycero-3-phosphoethanolamine-*N*-[4-(*p*-maleimidophenyl) butyramide (maleimide-headgroup lipid, MPB-PE). Carboplatin and paclitaxel were purchased from Sigma-Aldrich (St. Louis, MO).

BALB/c and athymic mice (Charles River Laboratories) were used for *in vivo* toxicity and anti-tumor studies. Mice were held under specific pathogen-reduced conditions in the Animal Facility of the University of Southern California (Los Angeles, CA, USA). All experiments were performed in accordance with the guidelines set by the National Institute of Health and were approved by the Institutional Animal Care and Use Committee of the University of Southern California.

### Preparation of cMLVs

Liposomes were prepared based on the conventional dehydration–rehydration method. All lipids were combined in chloroform, at a molar lipid ratio of DOPC–DOPG–MPB = 4 : 1 : 5, and the chloroform in the lipid mixture was evaporated under argon gas. The lipid mixture was further dried under vacuum overnight to form dried thin lipid films. In order to prepare cMLVs (CPT), cMLVs (PTX) and cMLVs (CPT + PTX), paclitaxel was mixed with the lipid mixture in organic solvent before formation of the dried thin lipid films. The resultant dried film was hydrated in 10 mM bis–tris propane at pH 7.0 with carboplatin by vigorous vortexing every 10 min for 1 h. Four cycles of 15 s sonication were then applied (Misonix Microson XL2000, Farmingdale, NY) on ice at 1 min intervals for each cycle. To induce divalent-triggered vesicle fusion, MgCl_2_ was added at a final concentration of 10 mM. The resulting multilamellar vesicles were further cross-linked by addition of dithiothreitol (DTT, Sigma-Aldrich), at a final concentration of 1.5 mM for 1 h at 37 °C. The resulting vesicles were collected by centrifugation at 14 000*g* for 4 min and then washed twice with phosphate-buffered saline (PBS). For pegylation of cMLVs, the particles were incubated with 1 μmol of 2 kDa PEG-SH (Laysan Bio Inc. Arab, AL) for 1 h at 37 °C. The particles were then centrifuged and washed twice with PBS. The final products were stored in PBS at 4 °C.

### Characterization of cMLVs

The hydrodynamic size of cMLVs was measured by dynamic light scattering (Wyatt Technology, Santa Barbara, CA). The particles were suspended in filtered water, vortexed and sonicated prior to analysis.

### *In vitro* encapsulation and release profiles

Concentrations of CPT and PTX were then measured by C-18 reverse-phase high-performance liquid chromatography (RP-HPLC) (Beckman Coulter, Brea, CA) at 227 nm. To study the encapsulation capacity of cMLVs corresponding to CPT, cMLVs (CPT) and cMLVs (CPT + PTX) were collected and washed twice with PBS, followed by lipid extraction of vesicles with 1% Triton X-100 treatment. To determine the encapsulation capacity of cMLVs with respect to PTX, the cMLVs (PTX) and cMLVs (CPT + PTX) suspensions were diluted by adding water and acetonitrile for a total volume of 0.5 mL. Extraction of paclitaxel was accomplished by adding 5 mL of *tert*-butyl methyl ether and vortex-mixing the sample for 1 min. The mixtures were centrifuged, and the organic layer was transferred into a glass tube and evaporated to dryness under argon. Buffer A (95% water, 5% acetonitrile) was used to rehydrate the glass tube. To test CPT and PTX concentration, 1 mL of the solution was injected into a C18 column, and CPT and PTX were detected at 227 nm after different retention times (flow rate 1 mL min^–1^). To obtain the release behavior of CPT and PTX from cMLVs, the releasing media was removed from cMLVs incubated in 10% FBS-containing media at 37 °C and replaced with fresh media daily. The concentration of CPT and PTX in the removed media was quantified by HPLC.

### *In vitro* cytotoxicity and data analysis

To evaluate the cytotoxicity, the viability of cell treated by drug-loaded cMLVs was assessed using the Cell Proliferation Kit II (XTT assay) from Roche Applied Science according to the manufacturer's instruction.

The OVCAR8 and NCI/ADR-RES cells in 100 μL of 10% FBS-containing media were seeded in the well (5 × 10^3^ cells per well) of a 96-well plate and incubated at 37 °C for 6 h. The cells were then exposed to a series of concentrations of free drugs and drug-loaded cMLVs, at different molar ratios of combined drugs for 48 h. 50 μL of XTT labeling mixture (Roche Applied Science) was then added. After incubation at 37 °C for 4 h, the absorbance of the solution was measured at 570 nm using a microplate reader (Molecular Devices) to determine the OD value. The data was given as mean ± standard deviation (SD) based on 3 independent measurements. The cell viability was calculated by subtracting absorbance values obtained from media-only wells from drug-treated wells and then normalizing to the control cells without drugs.^26^ The cell viability was calculated as follows: cell viability = OD treated/OD control × 100%, where OD treated was obtained from the cells treated by a particular agent, and OD control was obtained from the cells without any treatments. The fraction of cells affected (fa) at each drug concentration was subsequently determined for each well. The data was analyzed by nonlinear regression to get the IC_50_ value. The median-effect method assesses the drug–drug interaction by a term called the “combination index” (CI), which is based on the concentration–response relationship. CI was calculated by the equation: CI = D1/Dm1+D2/Dm2,^20^ where D1 and D2 are the doses of drug 1 and drug 2 that in combination produce some specified effect (*e.g.*, 50% inhibition of cells), and Dm1 and Dm2 are the drug doses that have the same effect when administered singly. The CI values lower than, equal to, and higher than 1 denote synergism, additivity, and antagonism, respectively.

### Flow cytometric analysis

FACS analysis was performed MACS Quant analyzer (Miltenyi Biotec Inc., San Diego, CA). OVCAR8 and NCI/ADR-RES cells were seeded into 6-well plates at 6 × 10^6^ cells per well. When cells reached about 70% confluence, they were incubated with PBS control, cMLVs (10 μM CPT), cMLVs (10 μM PTX), cMLVs (8 μM CPT + 2 μM PTX), cMLVs (5 μM CPT + 5 μM PTX) and cMLVs (2 μM CPT + 8 μM PTX) for 24 h. For ALDH staining, OVCAR8 and NCI/ADR-RES were harvested, and an aldefluor kit was used according to the manufacturer's instructions (Stem Cell Technologies, Vancouver, Canada). For analysis of autofluorescent subpopulations, OVCAR8 and NCI/RES-ADR cells were harvested and detected using SORP LSR II (BD Biosciences, San Jose, CA). Autofluorescent cells are excited with a 488 nm blue laser and best selected as the intersection with filters 525/50 and 575/26. A proper distance between gates for autofluorescent and non-autofluorescent cells is required.^27^

### Evaluation of the acute toxicity

Six-week-old female BALB/c mice were randomized into 5 groups (*n* = 3) based on body weight. All the mice were administered with PBS, CPT + PTX (10 mg + 20 mg kg^–1^ and 15 mg + 30 mg kg^–1^) and cMLVs (CPT + PTX) (10 mg + 20 mg kg^–1^ and 15 mg + 30 mg kg^–1^) through intravenous injection once. After the injection, the body weight and physical states of the mice were monitored every day. On day 7 after injection, animals were euthanized *via* CO_2_ overdose, livers were harvested and fixed in 4% paraformaldehyde. Then livers and kidneys were frozen and cut into sections and stained with hematoxylin and eosin (H&E) for pathology analysis.

### *In vivo* antitumor activity

Athymic mice were inoculated subcutaneously with 5 × 10^5^ OVCAR-8 cells in logarithmic growth phase from cell culture. Tumor volume was calculated according to the formula tumor volume (in millimeters cubed) = *D* × *d* ^2^/2, where *D* and *d* are the longest and shortest diameters, respectively. The tumors were allowed to grow for 2 month to a volume of ∼100 mm^3^ before treatment. The mice were injected intravenously through the tail vein with PBS, cMLVs (1.8 mg kg^–1^ CPT), cMLVs (4 mg kg^–1^ PTX), cMLVs (1.8 mg kg^–1^ CPT + 4 mg kg^–1^ PTX) every 3 days for four rounds (four mice per group). Tumor growth and body weight were monitored until the end of the experiment. The length and width of the tumor masses were measured with a fine caliper every 3 days after injection.

### Immunohistochemistry of tumors and confocal imaging

OVCAR8 ovarian tumors were harvested, fixed, frozen, and sectioned. Frozen sections were treated using the *In Situ* Cell Death Detection kit (Roche, Indianapolis, Indiana) as recommended by the manufacturers. Fluorescence images were taken using a Yokogawa confocal scanner system (Solamere Technology Group, Salt Lake City, UT) with a Nikon Eclipse Ti-E microscope. Illumination powers at 405, 491, 561, and 640 nm solid-state laser lines were provided by an AOTF (acousto-optical tunable filter)-controlled laser-merge system with 50 mW for each laser.

### Statistics

Data is presented as mean ± standard error (SEM). Statistical analyses to compare two groups and multiple groups were performed by student's *t*-test and one-way analysis of variance (ANOVA) respectively.

## Results and discussions

### Drug encapsulation and release from cMLVs

**Scheme 1:**
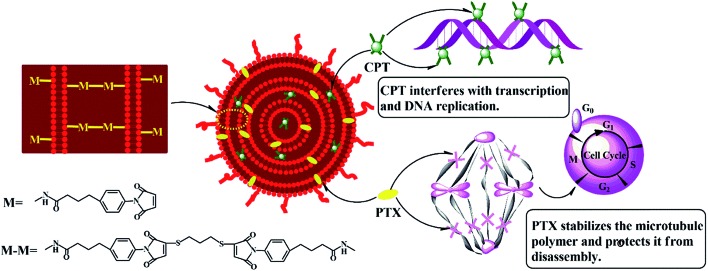
Schematic illustration of the codelivery of carboplatin and paclitaxel *via* cMLVs.

As shown in Scheme 1, the hydrophilic drug, carboplatin and hydrophobic drug, paclitaxel were encapsulated into the aqueous core and lipid membranes of cMLVs respectively. As a result, cMLVs could encapsulate binary therapeutic drugs with very different chemo-physical properties and mechanisms of action into a single nanocarrier, so as to synergistically enhance the efficacy of treatment. Furthermore, cMLVs' dispersion could be stabilized by PEG-coating and neutral zeta potential (Table S1†) which minimizes the interactions between the cMLVs and blood components.

As shown in Table 1, the single drug encapsulation capacity for CPT and PTX were 13.2 w/w% and 29.3 w/w% respectively. Interestingly, the binary drug-loaded cMLVs showed similar encapsulation capacities compared to their single drug-loaded formulation. Moreover, Table 1 shows that there is no significant difference in size between single drug-loaded cMLVs and binary drug-loaded cMLVs. These results could be explained by the fact that different compartments of cMLVs were occupied by CPT and PTX. Therefore, we have confirmed that loading multiple drugs into cMLVs has no effect on the encapsulation capacity of the individual drugs. In addition, sustained release behaviors were obtained from cMLV for both CPT and PTX (Fig. S1†).

**Table 1:** Drug encapsulation properties and mean diameter of cMLVs

Formulation	Encapsulation capacity (w/w%)		Diameter (nm)	PDI
	CPT	PTX		
cMLVs (CPT)	13.2 ± 0.3	—	206 ± 9.68	0.10
cMLVs (PTX)	—	29.3 ± 0.7	212 ± 8.13	0.08
cMLVs (CPT + PTX)	12.8 ± 0.2	28.7 ± 0.5	225 ± 10.97	0.11

### *In vitro* cytotoxicity of drug-loaded cMLVs

**Fig. 1:**
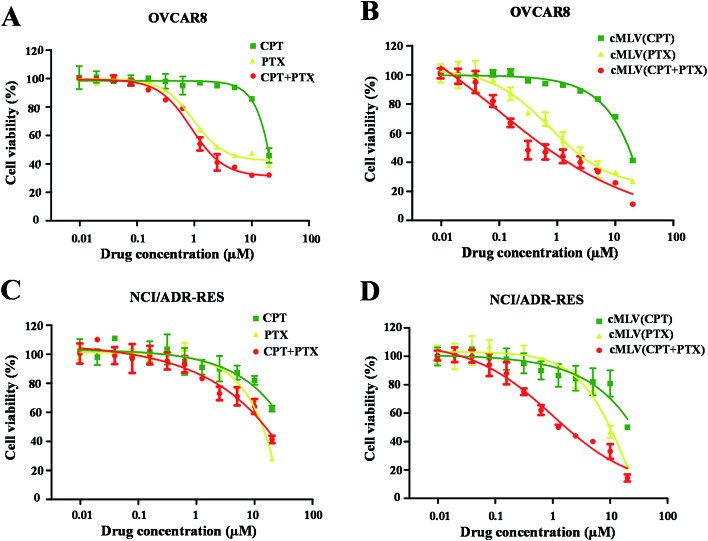
*In vitro* cytotoxicity profiles of CPT/PTX and cMLVs (CPT/PTX) against OVCAR8 (A and B) and NCI/ADR-RES (C and D) cells.

The cytotoxicity of the free drugs and their cMLV formulations was determined in human ovarian carcinoma OVCAR8 cells with an XTT assay. Because previous studies have reported that the drug combination could have a positive effect on overcoming multi-drug resistance in cancer cells,^5,28^ this study also reports the apoptotic effects of each treatment regimen on drug resistant, NCI/ADR RES cells. All the calculated IC_50_ values are summarized in Table 2. As shown in Table 2 and Fig. 1A and B, the single drug PTX (IC_50_, 5.85 μM, 9.86 μM) is more potent at inducing apoptosis than the single drug CPT (IC_50_, 21.09 μM, 79.36 μM) against OVCAR8 and NCI/ADR-RES cells. Afterwards, the anticancer efficacies of the free CPT + PTX drug combination and cMLVs (CPT + PTX) were evaluated. Both CPT + PTX (Fig. 1A and C) and cMLVs (CPT + PTX) (Fig. 1B and D) exhibited an enhancement of combination potency. The IC_50_s of free CPT alone and free PTX alone against OVCAR8 were 21.09 μM and 5.85 μM respectively, while the IC_50_s of CPT (1.52 μM) and PTX (1.52 μM) in free CPT + PTX combination were both reduced. Moreover, in contrast to the IC_50_s of cMLVs (CPT) (16.54 μM) and cMLVs (PTX) (2.69 μM) against OVCAR8, the IC_50_s of CPT and PTX in the cMLVs (CPT + PTX) formulation were reduced even more significantly. These data suggest synergistic cytotoxicity in free CPT + PTX combination against OVCAR8 cells, and the synergistic effect is more evident when the component drugs are encapsulated into one nanocarrier due to the nanocarrier's ability to ensure that both of the component drugs are delivered into the cell at a desired ratio.^25^ A similar synergistic effect was also observed for NCI/ADR-RES cells. Importantly, as shown in Fig. 1, the CPT + PTX combination delivery *via* cMLVs exhibits improved potency compared to the free CPT + PTX combination against OVCAR8 and NCI/ADR-RES cells. The enhanced synergism of cMLVs (CPT + PTX) is attributed to the cMLVs' morphology since their rate of endocytosis is often more efficient than the rate of passive diffusion for small molecular drugs.^29^ Additionally, it has been determined that the drug unloaded cMLVs do not exhibit any cytotoxicity to both OVCAR8 and NCI/ADR-RES (Fig. S2†).

**Table 2:** IC_50_ and CI_50_ values of free drug and cMLVs therapeutics against OVCAR8 and NCI/ADR-RES

Formulation CPT/PTX (molar ratio)	OVCAR8		NCI/ADR-RES	
	IC_50_ (μM)	CI50	IC_50_ (μM)	CI50
CPT	21.09	—	79.36	—
PTX	5.85	—	9.86	—
CPT + PTX	1.52/1.52	0.33	7.13/7.13	0.81
cMLVs (CPT)	16.54	—	18.88	—
cMLVs (PTX)	2.69	—	8.44	—
cMLVs (4CPT + PTX)	0.92/0.23	0.14	6.05/1.51	0.49
cMLVs (CPT + PTX)	0.29/0.29	0.13	1.65/1.65	0.28
cMLVs (CPT + 4PTX)	0.16/0.63	0.24	0.93/3.73	0.50

**Fig. 2:**
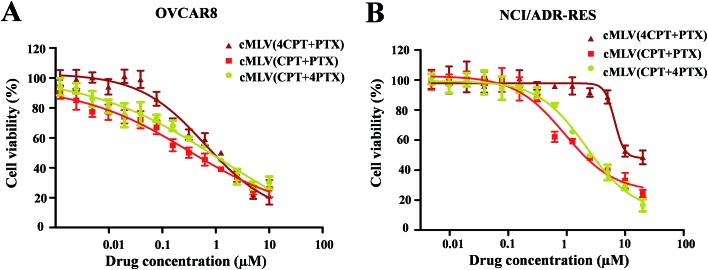
*In vitro* cytotoxicity profiles of cMLVs (CPT/PTX) with various CPT/PTX ratios against OVCAR8 (A) and NCI/ADR-RES (B) cells.

It has been widely reported that the dose ratio of the drug combination plays a very important role in the therapy's combination effect, synergy, additivity, and antagonism.^30^ In order to determine the optimal ratio that can induce the strongest synergy, the cytotoxicity of cMLV-encapsulated CPT and PTX combinations at three different molar ratios (CPT : PTX, 1 : 4, 1 : 1, 4 : 1) were assessed in OVCAR8 and NCI/ADR-RES cells. Table 2 displays the IC_50_ of the binary drugs-loaded cMLVs at the three different molar ratios. As shown in Fig. 2, the cytotoxicity of cMLVs (CPT/PTX) with CPT/PTX ratios at 1 : 4 and 1 : 1 were significantly stronger than that of the 4 : 1 molar ratio in both OVCAR8 and NCI/ADR-RES cells. This result is in accordance with a previous study,^23^ in which paclitaxel has a stronger cytotoxicity than a platinum-based drug against ovarian carcinoma cells.

To further study the synergy in cMLVs (CPT/PTX), we determined its combination index (CI). CI values lower than, equal to, and higher than 1 indicate synergism, additivity, and antagonism, respectively. As shown in Table 2, at 50% cell killing effect (CI_50_), synergistic effects were observed in both OVCAR8 and NCI/ADR-RES cancer cells treated with cMLVs (CPT/PTX) with three different molar ratios. However, the strongest synergistic cytotoxicity was observed at a 1 : 1 molar ratio of CPT/PTX in the co-loading cMLVs formulation showing the lowest CI_50_ values of 0.13 and 0.28 on OVCAR8 and NCI/ADR-RES cells respectively.

**Fig. 3:**
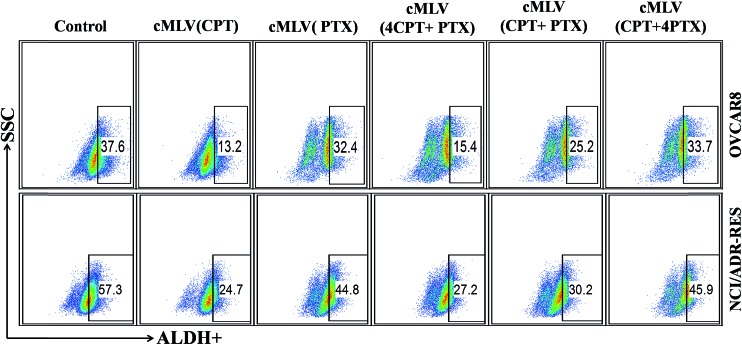
ALDH+ cells in unsorted OVCAR8 and NCI/ADR-RES cell lines determined by FACS analyses using ALDH activity assay.

It has been reported that ovarian cancer stem cells (CSCs) associates with recurrence in early-stage ovarian cancer.^31^ In addition, aldehyde dehydrogenase activity (ALDH+) is a known CSC biomarker for all human ovarian cancers.^32^ This prompted us to investigate the ability of drug-loaded cMLVs to eliminate CSCs in OVCAR8 and NCI/ADR-RES cell lines. FACS analyses showed that untreated OVCAR8 and NCI/ADR-RES cell lines contained 37.6% and 57.3% ALDH+ cells, respectively (Fig. 3). Ovarian cancer cells treated by cMLVs (CPT) reduced the population of ALDH+ cells significantly in OVCAR8 (13.2%) and NCI/ADR-RES (24.7%), whereas treatment of cMLVs (PTX) did not show a strong ability to alter ALDH+ population (32.4% and 44.8%, in OVCAR8 and NCI/ADR-RES cells respectively) (Fig. 3). The stronger cytotoxicity of CPT against CSCs could be explained by the different mechanisms of action of PTX and CPT. PTX is an M-phase specific antitumor drug and acts on highly proliferative cells. However, it has been reported that the cancer stem cells exhibit a quiescent slow-cycling phenotype.^33,34^ Thus, PTX is not able to kill the cancer stem cells efficiently. This is also the reason why cancer stem cells can induce resistance to conventional chemotherapies that are dependent on cell cycle inhibition.^35^ In contrast, cisplatin is a cell cycle independent antitumor drug that covalently binds to DNA and leads to efficient elimination of CSCs. The combination treatments using cMLVs with different ratios of CPT/PTX also showed different effectiveness at inhibiting the growth of ALDH+ populations in OVCAR8 and NCI/ADR-RES cells. As shown in Fig. 3, the ability of cMLVs (CPT/PTX) at molar ratios of CPT/PTX of 4 : 1 and 1 : 1 to eliminate ALDH+ cells was significantly stronger than that of cMLVs (CPT/PTX) prepared at 1 : 4 molar ratio of CPT/PTX, in both OVCAR8 and NCI/ADR-RES cells. However, *in vitro* cytotoxicity assays showed that cMLVs (CPT/PTX) at CPT/PTX ratios of 1 : 4 and 1 : 1 displayed stronger cytotoxicity in OVCAR8 and NCI/ADR-RES cell lines. Taken together, these results indicate that 1 : 1 is the desired molar ratio for cMLVs (CPT/PTX) combination treatment to target the bulk and ALDH+ CSCs population. Hence, all the CPT and PTX loaded cMLVs were prepared at 1 : 1 molar ratio of CPT/PTX in the following studies.

### *In vivo* toxicity

To determine whether cMLVs could reduce systemic toxicity, the acute toxicity of the combination therapeutics was studied. According to previously published data, the maximum tolerated dose of free PTX for cancer treatments was set at 20 mg kg^–1^ in mice models.^36^ Thus, we tested the acute toxicity of the free drug combination (CPT + PTX) and cMLVs (CPT + PTX) with a PTX dosage of 20 mg kg^–1^ and 30 mg kg^–1^. These treatments were injected into BALB/c mice intravenously through the tail vein. The weights and overall behavior of the mice were monitored for 7 days after the single injection.

**Fig. 4:**
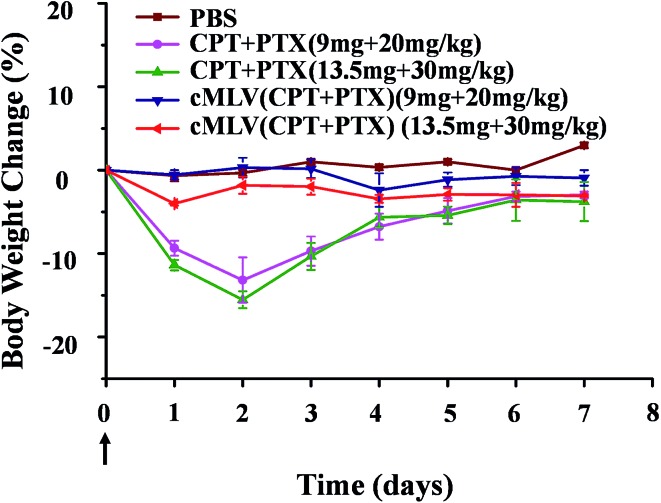
The body weight changes of BALB/c mice treated with a single dose of free drug mixture of CPT + PTX and cMLVs (CPT + PTX) at 20 and 30 mg PTX per kg levels in comparison with PBS control group. Error bars represent standard error of the mean, *n* = 3 for each treatment group.

On day 2, as shown in Fig. 4, the groups treated with the free drug combinations at 20 and 30 mg kg^–1^ of PTX dosage experienced a loss of 13.2 and 15.5% of their body weight respectively. In contrast, the two cMLVs (CPT + PTX) administration groups showed a much smaller body weight loss (2–4%). It was noted that all mice receiving the free drug combination at all dose levels showed convulsions over 2 minutes after injection, whereas no convulsions were observed with all dose levels of nanotherapeutics. There was no difference in the general behavior between mice in the cMLVs (CPT + PTX) groups and mice in the PBS group in the following days, but the mice treated with free drug combinations were significantly less active.

**Fig. 5:**
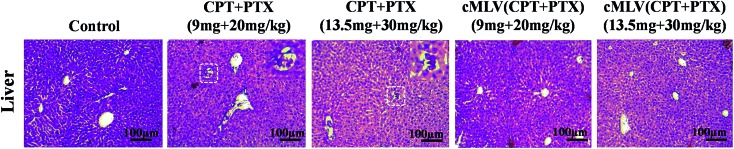
Histopathological changes in liver from the acute toxicity studies in BALB/c mice on day 7 after one intravenous treatment with PBS, free drug mixture of CPT + PTX and cMLVs (CPT + PTX) at 20 and 30 mg PTX per kg levels in comparison with PBS control group.

Light microscopic examination of H&E stained liver sections from sacrificed animals showed that liver had no morphological changes after the treatment with cMLVs (CPT + PTX) compared with the control group, while obstruction of sinusoids in livers were observed from all animals treated with the free drug combination (Fig. 5). The reduced systemic toxicity of cMLVs (CPT + PTX) could be attributed to its macromolecular size and slow drug release rate.

### *In vivo* anticancer efficacy of drug-loaded cMLVs

We evaluated the efficacy of our cMLVs (CPT + PTX) combination therapy using OVCAR8 xenograft nude mice. When tumors were about 100 mm^3^ in size, the mice were randomly divided into 4 groups and were given four injections of drug-loaded cMLVs intravenously on days 0, 3, 6, and 9, where day 0 is the day of the first injection. The tumor volume and body weight were then monitored every three days for 12 days. The treatments for the 4 groups were as follows: (a) PBS control, (b) cMLVs (CPT) (1.8 mg kg^–1^), (c) cMLVs (PTX) (4.0 mg kg^–1^) and (d) cMLVs (CPT + PTX) (1.8 mg + 4.0 mg kg^–1^). No weight change was observed in all treatment groups for the duration of the experiment, denoting the absence of systemic toxicity from the cMLVs nanotherapeutics.

**Fig. 6:**
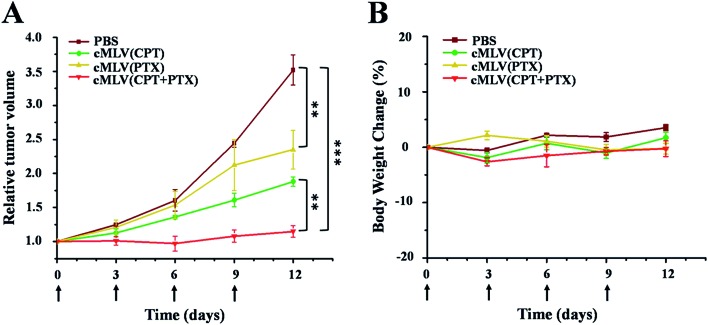
*In vivo* tumor growth inhibition (A) and body weight changes (B) of mice bearing OVCAR8 ovarian cancer xenografts after intravenous treatment with PBS, cMLVs (CPT) (1.8 mg kg^–1^), cMLVs (PTX) (4.0 mg kg^–1^) and cMLVs (CPT + PTX) (1.8 mg kg^–1^ + 4.0 mg kg^–1^) on days 0, 3, 6 and 9. Error bars represent standard error of the mean, *n* = 4 for each treatment group (**p < 0.01; ***p < 0.001).

Meanwhile, in terms of tumor inhibition, mild tumor inhibitory effects (*p* < 0.01) were exhibited by cMLVs (CPT) (1.8 mg CPT per kg) and cMLVs (PTX) (4.0 mg PTX per kg) compared to the control group (Fig. 6). However, no differences in tumor growth inhibition (*p* = 0.09) were seen between cMLVs (CPT) (1.8 mg CPT per kg) and cMLVs (PTX) (4.0 mg PTX per kg). More importantly, cMLVs (CPT + PTX) (1.8 mg CPT + 4.0 mg PTX per kg) displayed stronger tumor inhibitory effects (*p* < 0.01) than both cMLVs (CPT) (1.8 mg CPT per kg) and cMLVs (PTX) (4.0 mg PTX per kg). On day 12, the relative tumor volume median for cMLVs (CPT + PTX) (1.8 mg CPT + 4.0 mg PTX per kg) was 1.1. Comparatively, the relative tumor volume median for mice treated with cMLVs (CPT) (1.8 mg CPT per kg), cMLVs (PTX) (4.0 mg PTX per kg) and PBS were 2.0, 1.9 and 3.5, respectively. The superior tumor inhibition of cMLVs (CPT + PTX) further confirms the synergistic effects between CPT and PTX.

**Fig. 7:**
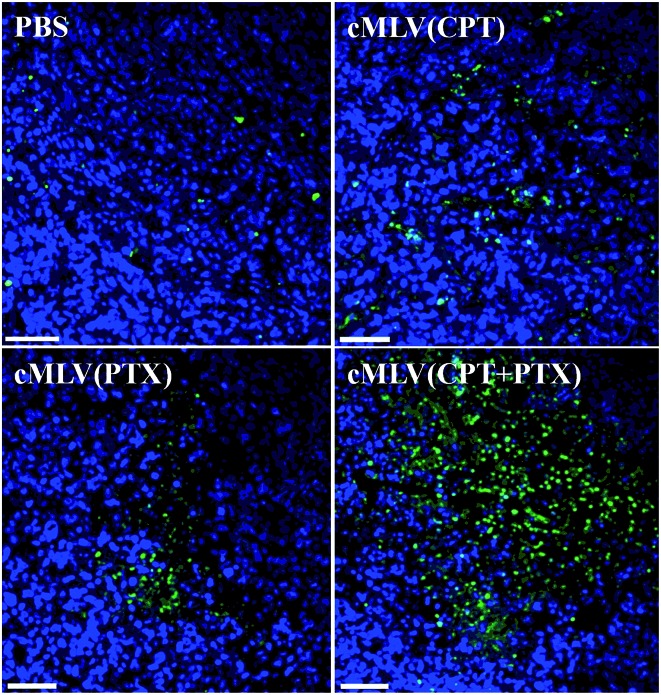
TUNEL staining of apoptotic positive cells in the OVCAR8 tumor. Mice beard OVCAR8 ovarian cancer xenografts were treated with PBS, cMLVs (CPT) (1.8 mg kg^–1^), cMLVs (PTX) (4.0 mg kg^–1^) and cMLVs (CPT + PTX) (1.8 mg kg^–1^ + 4.0 mg kg^–1^) on days 0, 3, 6 and 9. On day 12, tumors were excised. Apoptotic cells were detected by a TUNEL assay (green) and costained by nuclear staining DAPI (blue). The scale bar represents 50 μm.

A TUNEL assay was then conducted to observe the number of apoptotic cells in OVCAR8 tumors treated with CPT and PTX and CPT + PTX in cMLV formulations for 4 rounds, in order to further investigate whether the synergistic effects could also be induced by the *in vivo* co-delivery of CPT and PTX *via* cMLVs. As shown in Fig. 7, OVCAR8 tumors treated with cMLVs (CPT) and cMLVs (PTX) induced a moderate amount of cell apoptosis compared to the controls. Additionally, cMLVs (CPT + PTX) promoted a more remarkable tumor cell apoptosis than both cMLVs (CPT) and cMLVs (PTX). These results are consistent with results of the inhibitory effect on tumor growth (Fig. 6).

## Conclusions

In this study, we have utilized a cross-linked multilamellar liposome for the effective co-delivery of CPT and PTX as an ovarian cancer combination therapy. These multi-compartment and cross-linked nanostructures enable the highly encapsulation efficacy for both CPT and PTX. When combined at the optimal drug-loading ratio, the different mechanisms of action of CPT and PTX allow for synergistic, cytotoxic effects against ovarian cancer cells. *In vivo* studies showed that the binary drug combination therapy administrated *via* cMLVs displayed a safe and effective inhibition toward OVCAR8 tumor growth. Overall, this CPT and PTX co-delivery system has potential for use in a clinical setting as an ovarian cancer treatment.
