# Supplementary material for: Co-delivery of carboplatin and paclitaxel via cross-linked multilamellar liposomes for ovarian cancer treatment
Source: RSC Adv. 2017 Apr 3;7(32):19685–93. doi: 10.1039/c7ra01100h (PMC5450007; doi:10.1039/c7ra01100h)
Supplement: Supplementary file 1 [file RA-007-C7RA01100H-s001.pdf]

Electronic Supplementary Material (ESI) for RSC Advances.
This journal is © The Royal Society of Chemistry 2017

Co-delivery of Carboplatin and Paclitaxel via Cross-linked Multilamellar Liposome for Ovarian Cancer Treatment

Supplementary Information

TableS1. Zeta potential of cMLVs.

Formulation	cMLV	cMLV(CPT)	cMLV(PTX)	cMLV(CPT+PTX)
Zeta Potential(mV)	-3.76±0.17	-3.51±0.18	-3.64±0.18	-3.63±0.29

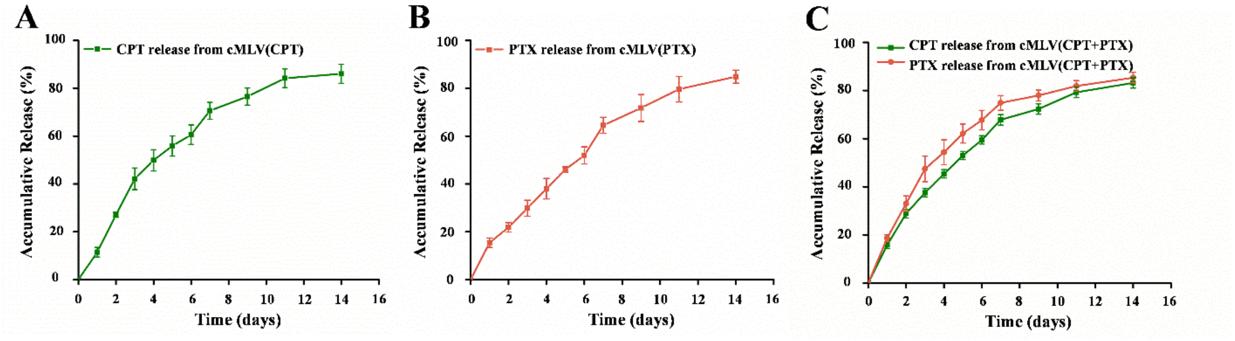

Fig. S1 In vitro release profiles of carboplatin and paclitaxel from cMLV(CPT) (A), cMLV(PTX)(B) and cMLVs(CPT+PTX) (C). Error bars represent the standard deviation of the mean from triplicate experiments.

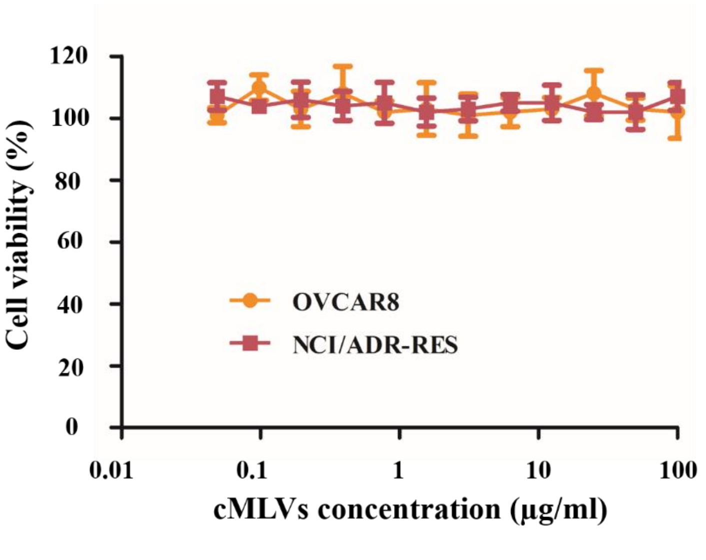

Fig. S2 In vitro cytotoxicity profiles of cMLVs against OVCAR8 and NCI/ADR-RES cells.
